# Methanogenic Archaea Can Produce Methane in Deliquescence-Driven Mars Analog Environments

**DOI:** 10.1038/s41598-019-56267-4

**Published:** 2020-01-08

**Authors:** Deborah Maus, Jacob Heinz, Janosch Schirmack, Alessandro Airo, Samuel P. Kounaves, Dirk Wagner, Dirk Schulze-Makuch

**Affiliations:** 1 Zentrum für Astronomie und Astrophysik (ZAA), AG Astrobiologie, Technische Universität Berlin, Hardenbergstr. 36, 10623 Berlin, Germany; 2 Metabolism of Microbial Pathogens, Robert Koch-Institute, Berlin, Germany; 3 Department of Chemistry, Tufts University, Medford, Massachusetts USA; 4 Department of Earth Science and Engineering, Imperial College, London, UK; 5 GFZ German Research Center for Geosciences, Section Geomicrobiology, Potsdam, Germany; 6 Institute of Geosciences, University of Potsdam, Potsdam, Germany; 7 Leibniz-Institute of Freshwater Ecology and Inland Fisheries (IGB), Department of Experimental Limnology, Stechlin, Germany

**Keywords:** Microbial ecology, Astrobiology

## Abstract

The current understanding of the Martian surface indicates that briny environments at the near-surface are temporarily possible, e.g. in the case of the presumably deliquescence-driven Recurring Slope Lineae (RSL). However, whether such dynamic environments are habitable for terrestrial organisms remains poorly understood. This hypothesis was tested by developing a Closed Deliquescence System (CDS) consisting of a mixture of desiccated Martian Regolith Analog (MRA) substrate, salts, and microbial cells, which over the course of days became wetted through deliquescence. The methane produced via metabolic activity for three methanogenic archaea: *Methanosarcina mazei*, *M. barkeri* and *M. soligelidi*, was measured after exposing them to three different MRA substrates using either NaCl or NaClO_4_ as a hygroscopic salt. Our experiments showed that (1) *M. soligelidi* rapidly produced methane at 4 °C, (2) *M. barkeri* produced methane at 28 °C though not at 4 °C, (3) *M. mazei* was not metabolically reactivated through deliquescence, (4) none of the species produced methane in the presence of perchlorate, and (5) all species were metabolically most active in the phyllosilicate-containing MRA. These results emphasize the importance of the substrate, microbial species, salt, and temperature used in the experiments. Furthermore, we show here for the first time that water provided by deliquescence alone is sufficient to rehydrate methanogenic archaea and to reactivate their metabolism under conditions roughly analogous to the near-subsurface Martian environment.

## Introduction

Methane in the atmosphere of Mars was first detected by Formisano, *et al*.^1^ with the Planetary Fourier Spectrometer onboard the Mars Express orbiter. Recent measurements by the Tunable Laser Spectrometer onboard the Curiosity rover show that methane concentrations are currently in the parts per billion range and fluctuate repeatedly throughout the seasons^2^. However, the long-term stability and presence of methane in the Martian atmosphere is considered unlikely over geological time periods due to degradation by UV radiation and/or oxidizing compounds at the surface^3^. Hence, it has been proposed that subsurface reservoirs might sporadically release methane and/or that it is actively produced through abiotic, or potentially, as it is on Earth, from microbial processes^4^.

On Mars, however, the environmental conditions within the surficial regolith generally do not permit a lasting presence of liquid water necessary for microbial metabolism. Nevertheless, besides surficial monolayers of liquid-like water^5^ and morning frost^6^, a possible exception are near-surface environments laden with hygroscopic salts which can undergo deliquescence, a process occurring when the relative humidity (RH) exceeds the deliquescence relative humidity (DRH) of a given salt above its eutectic temperature. Such a mechanism was first hypothesized by McEwen, *et al*.^7^ to play a role in the seasonal surface features on Mars known as Recurring Slope Lineae (RSL), dark streaks that appear on steep crater walls situated in the mid-latitudes. Although some authors have questioned the role of liquid water for this phenomena^8^, others support the hypothesis of a deliquescence-driven transient brine formation at the RSLs^9,10^. Further support for this deliquescence-driven process is provided by the remote detection of chlorides at various locations on Mars^11^, the discovery of perchlorates at both, the Phoenix Lander and Curiosity Rover sites^12–14^, and the putative observation of deliquescence at the Phoenix Lander struts by Rennó, *et al*.^15^.

Analogous environments on Earth and the study of their associated microbiota can greatly aid in evaluating the habitability of such Martian environments. Arid and salt-laden environments subject to deliquescence and populated by adapted microbial communities are for example, the Atacama Desert, Chile^16^, the McMurdo Dry Valleys, Antarctica^6^, or the Larsemann Hills in East Antarctica^17^.

Extremophilic microorganisms adapted to such arid and briny environments show an enhanced survival in chloride and perchlorate-bearing brines at the respective eutectic salt concentrations when subjected at subzero temperatures^18,19^. Among those microorganisms that show adaptive behavior at low temperatures and high salinities are methanogenic archaea^20^. They can thrive on CO_2_ and H_2_ as a sole energy and carbon source while producing methane; a metabolic pathway known as methanogenesis. Previous studies on methanogens have shown that Martian soil simulants, i.e. Martian Regolith Analogs (MRAs), serve as suitable substrates for growth for these kind of microorganisms^21^ and enhance their survival during desiccation^22,23^.

**Figure 1:**
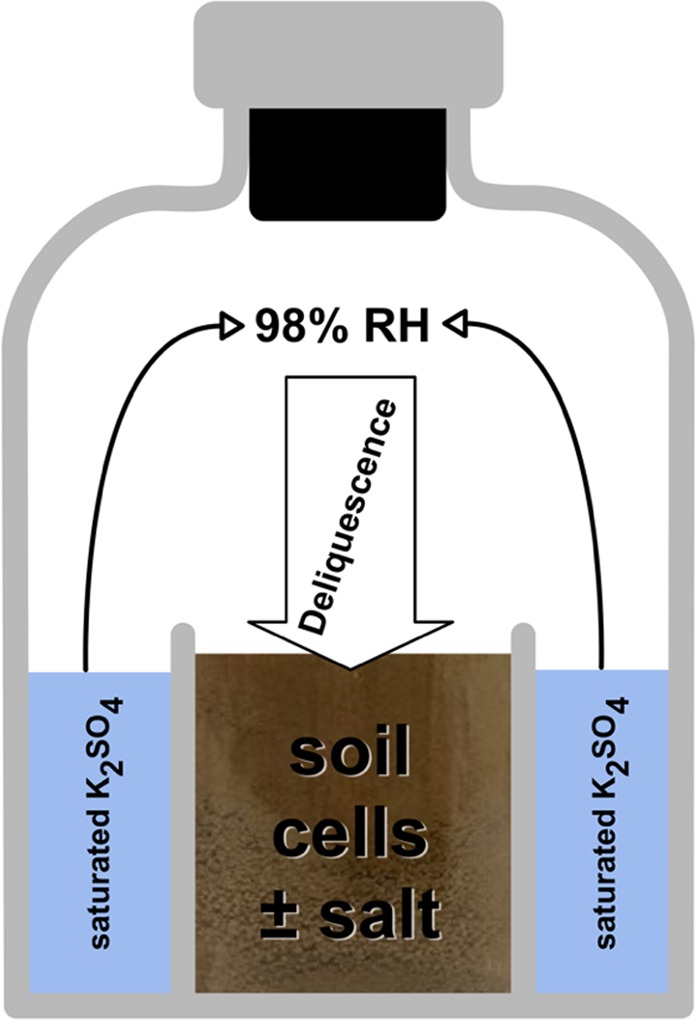
Experimental setup: The principal process of water transport within the Closed Deliquescence System. The positive control and the actual deliquescence experiment samples contained salts while the negative control samples did not contain salts.

Methanogenic archaea might be able to thrive in some Martian environments and could be a potential biogenic source for the methane in the atmosphere of Mars. Until now, research on methanogens as a model organism for evaluating the habitability of Martian environments has focused on stress factors such as desiccation, drought, starvation, freeze and thaw cycles, high salinity, low atmospheric pressure and elevated radiation dosages^20–26^. However, to our knowledge, there have been no studies reported showing if methanogenic archaea can survive in a near-subsurface environment where water is only provided by deliquescence. We report here on experiments, in a Closed Deliquescence System (CDS) using a Martian regolith analog (Fig. 1), that show methanogenic archaeal strains can regain metabolic activity after having been desiccated and subsequently wetted through *in vitro* deliquescence.

## Results

### Methane production of methanogenic archaea in the martian regolith analogs (MRAs)

**Figure 2:**
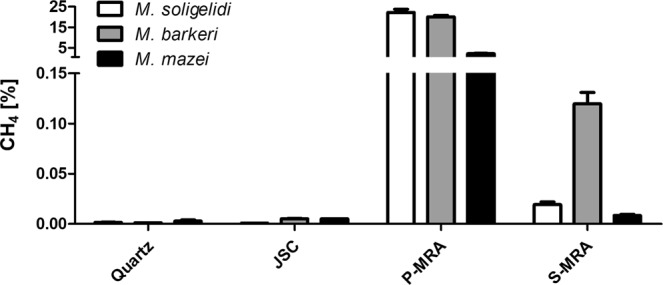
Substrate evaluation: cumulative methane production of methanogens after desiccation and rehydration in different Martian Regolith Analogs (no additional salts) after 21 days of incubation at 28 °C (n = 3, SEM).

Initially, we tested the ability of three *Methanosarcina* species mixed with different substrates (MRAs or quartz sand) to survive desiccation and to subsequently be reactivated by rehydration with pure water and produce methane (Fig. 2). After 21 days of incubation the methanogens did not produce any significant amounts of methane (<35 ppm) either when quartz sand or MRA JSC-1A (Martian Regolith Analog from the Johnson Space Center, see *Material and Methods* for details) was used as a substrate. In the case of S-MRA (Martian Regolith Analog containing sulfatic minerals, see *Material and Methods* for details), methane production was substantially higher as *M. soligelidi* produced 190 ppm (0.019%), *M. mazei* 140 ppm (0.014%), and *M. barkeri* 1190 ppm (0.119%) methane. If P-MRA (Martian Regolith Analog containing phyllosilicates, see *Material and Methods* for details) substrate was used, methane production was more than two orders of magnitude higher, reaching 22.2% (*M. soligelidi*), 20.1% (*M. barkeri*) and 2.2% (*M. mazei*).

### Deliquescence test

We monitored the occurrence of deliquescence within our CDS by weighing the amount of water transferred from the outer water reservoir through the headspace into the inner compartment containing the cell-salt-substrate mixture.

**Figure 3:**
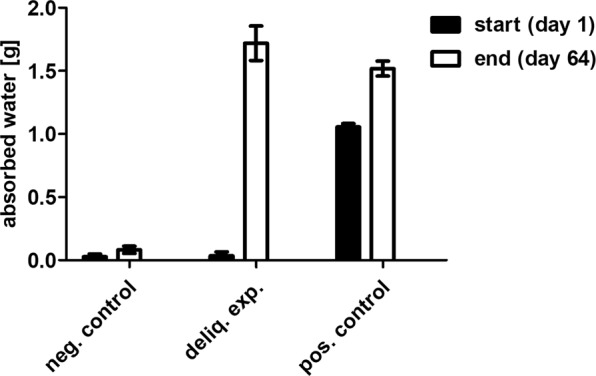
Deliquescence evaluation: water content of the inner compartment of the Closed Deliquescence System before and after 64 days at 4 °C with *M. soligelidi*, P-MRA and NaCl (n = 3, SEM).

The cumulative amount of water transferred after 64 days of incubation at 4 °C was 1.7 g for P-MRA containing NaCl, whereas the negative control lacking salt showed no significant change in weight (Fig. 3). The positive control containing NaCl and additionally 1 mL of water that was added at the beginning also increased in weight by 0.5 g, showing that deliquescence occurred. In general, the transfer of water was more rapid at 28 °C versus 4 °C, and with added NaClO_4_ instead of NaCl.

### Metabolic activity in the closed deliquescence system

The main purpose of the CDS was to test *in vitro* if methanogenic archaea can be metabolically reactivated through deliquescence (Table 1).

**Table 1:** Summary of the experiments performed in the Closed Deliquescence System: We incubated three species of methanogenic archaea with two types of Martian Regolith Analogs P-MRA (underlined, first column of each temperature and compound) and S-MRA (not underlined, second column) at two temperatures (4 °C and 28 °C) and with two salt species (NaCl and NaClO_4_).

Methanogenesis?	4 °C		28 °C		NaCl		NaClO_4_	
*M. soligelidi*	✓	nd	×	nd	×	nd	×	nd
*M. barkeri*	×	✓	✓	✓	✓	✓	×	×
*M. mazei*	×	×	×	×	×	×	×	×

A check mark indicates a condition in which methanogenesis was observed, a cross mark reflects a negative result. (nd = not determined).

**Figure 4:**
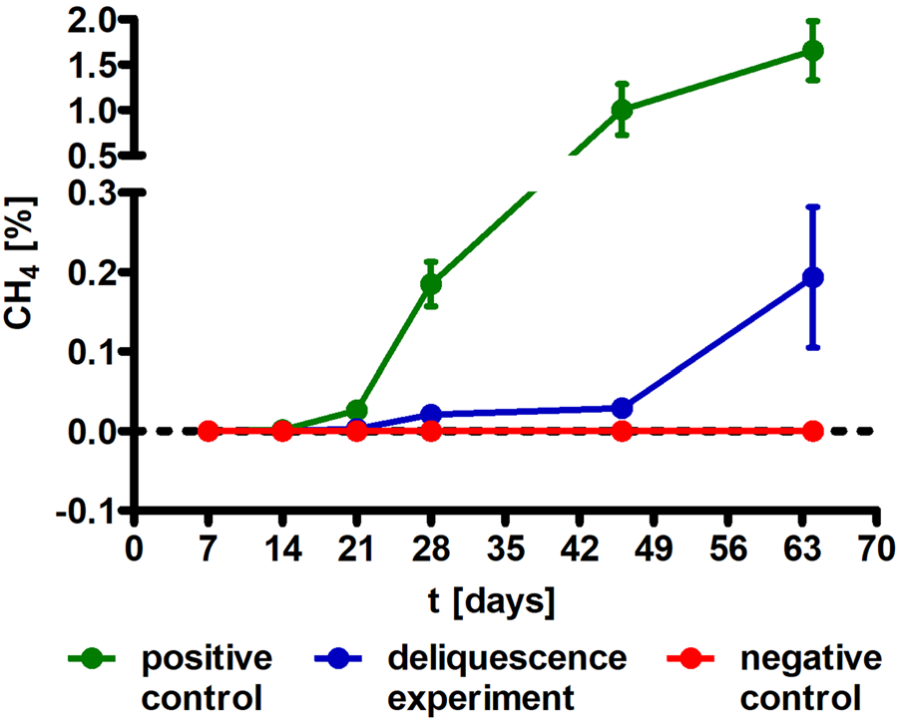
Methane content in the headspace observed as proxi for metabolic activity in the Closed Deliquescence System during the course of the experiment: *M. soligelidi* was tested in P-MRA with 30 wt% NaCl at 4 °C for 64 days (n = 3, SEM).

Our experiments indicated that *M. soligelidi* began producing methane after 28 days of incubation at 4 °C in P-MRA containing 30 wt% NaCl, reaching 0.19% methane after 64 days (Fig. 4). Earlier methane production was observed in the positive control (containing additional 1 mL of water) after 21 days and a higher final methane concentration of 1.65% after 64 days. The negative control (containing no additional salt) showed no significant methane production (<33 ppm). Although metabolic activity of *M. soligelidi* was present in the MRA evaluation experiments at 28 °C (see section 3.1), no metabolic activity could be detected in the deliquescence experiments at this temperature.

**Figure 5:**
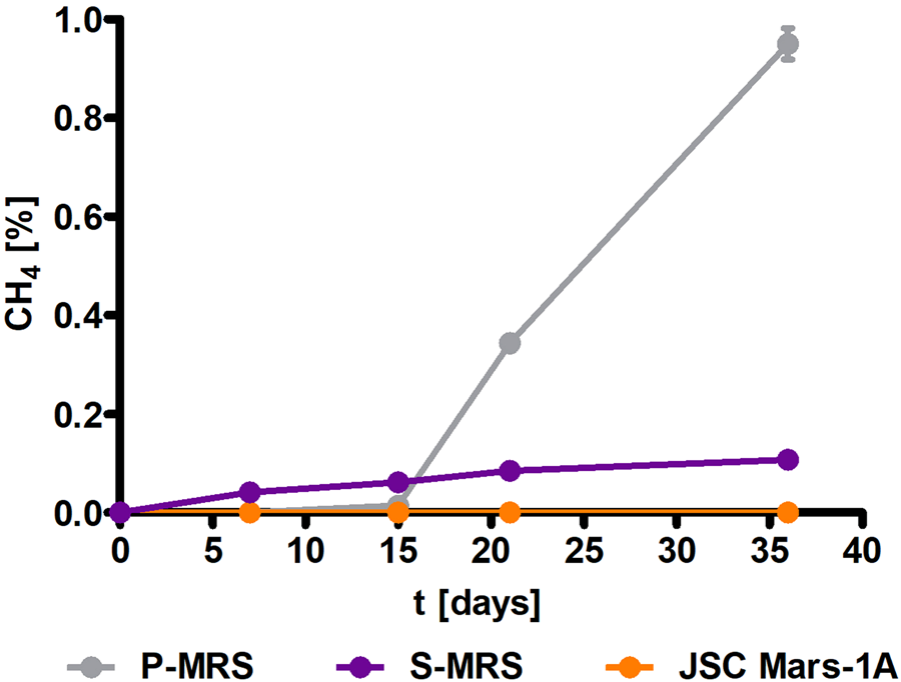
Metabolic activity in the Closed Deliquescence System with different substrates: *M. barkeri* was incubated at 28 °C in P-MRA, JSC Mars-1A or S-MRA, mixed with 30 wt% NaCl, for 36 days (n = 3, SEM).

In contrast, deliquescence experiments conducted with *M. barkeri* indicated no methane production at 4 °C but did at 28 °C. Furthermore, methane production was significantly different depending on the MRA used (Fig. 5). After 36 days of incubation at 28 °C, 0.95% methane was produced in P-MRA, while only 0.11% methane was produced in S-MRA. No significant accumulation of methane resulted when using JSC Mars-1A.

No methane production was observed in deliquescence experiments with *M. mazei* under any of the tested conditions nor did any of the experiments using NaClO_4_ (Table 1).

## Discussion

Our results show, for the first time, that rehydration of MRAs through deliquescence can reactivate the metabolism of desiccated methanogenic archaea (Fig. 4). The design of the CDS provided deliquescence-driven water transport into the incubation chamber (Fig. 1) but only when a hygroscopic salt was present. Our data also showed that methane production depends greatly on the methanogenic species, the incubation temperature, and the type of MRA and salt used in the experiments.

The choice of substrate had a major effect on the metabolic activity of the tested strains, where substantial methane production only occurred with P-MRA, while in S-MRA the production was roughly 2-orders-of-magnitude lower, and no significant activity was detected in JSC Mars-1A and quartz sand. It remains unclear why the P-MRA was most suitable for microbial metabolism, however, we suspect that the phyllosilicates, only present in P-MRA, may have played an essential role. Phyllosilicates, such as montmorillonites, have a high swelling capacity, an ability to retain small amounts of water protecting cells during desiccation, and have also been shown to serve as a nutrient source for methanogenic archaea^27^.

*M. mazei*, isolated from a sewage sludge in California and being phylogenetically the closest relative to *M. soligelidi*, did not show any significant methanogenic activity, presumably due to its inability to cope with either desiccation, high salinities, or the type of MRA.

If perchlorate was added to the MRAs, none of the tested organisms were metabolically reactivated and produced no methane. This is not surprising considering that perchlorate are known to inhibit metabolism at moderate to high concentrations^18^ and especially since at the beginning of the deliquescence process the forming salt solution is highly concentrated.

While *M. barkeri*, isolated from a sewage sludge in Illinois, was only metabolically active during the deliquescence experiments at an incubation temperature of 28 °C, *M. soligelidi*, isolated from permafrost soil in Siberia, was only metabolically active at 4 °C (although an optimal growth temperature of 28 °C has been reported^28^). However, considering that during our experiments the organisms had to cope with high salinities, our results are generally in agreement with previous findings showing that *M. soligelidi* has a higher salt tolerance at lower temperatures^20^.

Our results show that *M. soligelidi* is an especially suitable model organism for studying how microbial life could thrive in Martian environments that are subject to deliquescence producing conditions. Considering the UV radiation and freeze-thawing tolerance of *M. soligelidi*^24^, this organism is in principle well adapted to conditions expected to be prevalent within the salty shallow subsurface at RSL locations on Mars. Although UV radiation tolerance would not be necessary within the shallow subsurface, it would be crucial for aeolian-driven dispersion. Other studies have shown that methanogenic archaea can also withstand Mars-like conditions such as pressures of 50 to 400 mbar^26^ or three weeks of simulated Martian thermal conditions^20^.

Although *M. soligelidi* is a useful model organism for near surface habitats on Mars, such organisms could also thrive in deep subsurface environments. Aqueous environments, such as the putative subglacial lake near the Martian south pole^29^, could be supplied with H_2_ through serpentinization reactions, in turn feeding methanotrophs and generating methane. If not consumed otherwise, the long-term accumulation of methane and subsequent sporadic release could possibly explain the spikes in atmospheric methane measured from orbit and on the ground.

## Conclusion

With the custom-designed Closed Deliquescence System (CDS), we have simulated the *in vitro* process of deliquescence and demonstrated that *Methanosarcina soligelidi* and *Methanosarcina barkeri* can survive desiccation in the presence of 30 wt% sodium chloride in a phyllosilicate-containing Martian Regolith Analog, and furthermore become metabolically active after water is provided by deliquescence. Thus, we conclude that methanogenic archaea can live in environments with transient water availability and cope with varying salt concentrations. Consequently, salt-rich near subsurface environments on Mars subject to periodic wetting, such as proposed for RSLs, can be considered potential habitats for certain halotolerant methanogenic archaea and could potentially be a biological source of methane in the Martian atmosphere.

## Materials and Methods

### Microbial cultures

For this study, we used the methanogenic archaea *Methanosarcina soligelidi* SMA-21, isolated from the active layer of permafrost in the Lena Delta, Siberia^28^, and as reference organisms we used two non-permafrost derived strains: *Methanosarcina barkeri* DSM 8687 (obtained from the Leibniz Institute DSMZ-German Collection of Microorganisms and Cell Cultures), which was isolated in 1966 from a liquid sample of a domestic sewage sludge digestor in Urbana, Illinois^30^, and the phylogenetically closest relative to *M. soligelidi*, *Methanosarcina mazei* DSM 2053 ^T^ (also obtained from the DSMZ), isolated from a laboratory digester fed on urban wastes, raw domestic sewage sludge in El Segundo, California^31,32^. Cultures were incubated under anaerobic conditions at 28 °C in MW medium^33^ and a gas mixture of 100 kPa H_2_/CO_2_ (80:20 v/v) and 200 kPa N_2_/CO_2_ (80:20 v/v).

### Martian regolith analogs

We used Martian Regolith Analogs (MRAs) composed of basaltic rocks from Earth resembling Martian regolith. The MRA ‘JSC Mars-1A’ was produced at the Johnson Space Center, NASA, from Hawaiian volcanic deposits^34,35^. P- and S-MRA were developed by Jörg Fritz (Museum für Naturkunde, Berlin, Germany), which were previously tested with methanogens and described in detail by Schirmack, *et al*.^22^. In brief, P-MRA contains 45% montmorillonite, 20% chamosite, 10% quartz, and 25% other components, while S-MRA contains 31% gabbro, 30% gypsum, 17% hematite, 16% dunite, and 6% other components. Washed quartz sand with a mean grain size of 200 μm was used as a control substrate.

### Methane production by methanogenic archaea in martian regolith analogs

For testing metabolic activity within MRAs a 1 mL cell suspension (containing 10^8^ cells in MW medium) was mixed with 3 g of anoxic MRA and desiccated over calcium chloride for 72 hours in an anaerobic container after replacing the atmosphere three times with N_2_ ending with a reduced pressure of 300 to 400 mbar. After restoring the initial water content with anoxic water, the bottles were flushed with a gas mixture of 100 kPa H_2_/CO_2_ (80:20 v/v) and pressurized with 200 kPa N_2_/CO_2_ (80:20 v/v) and incubated at their respective temperature optimum (28 °C) for 21 days. A 250 µL gas sample of the headspace was taken at regular intervals with a syringe through the septum for determining the methane content by gas chromatography.

### Closed deliquescence system

The Closed Deliquescence System (CDS) is a glass vessel containing two separate compartments which share the same headspace (Fig. 1). The outer compartment is filled with a saturated potassium sulfate solution, assuring that the headspace has a stable relative humidity (RH) of 98%, which is as humid as possible without allowing water to condensate at the glass wall and possibly dripping into the inner compartment. This ensures a deliquescence driven water transport. The CDS was sealed with a rubber stopper and an aluminum crimp which function as a septum. The atmosphere was replaced with a gas mixture (flushed with 100 kPa H_2_/CO_2_ (80:20 v/v) and pressurized with 200 kPa N_2_/CO_2_ (80:20 v/v)) as described by Hungate^36^. The CDSs were incubated at either 28 °C or 4 °C and the methane content in the headspace was measured regularly through gas chromatography. The change in water content of the inner compartment of the CDS before and after the experiments was determined through weighing.

For the deliquescence experiments the inner compartment was filled with a desiccated mixture of 3 g MRA, 10^8^ cells (preparation described in 5.3), and 30 wt% salt (NaCl or NaClO_4_) to facilitate water transfer through the headspace driven by deliquescence. The negative control contained neither water nor salts and thus no deliquescence should occur. During the process of deliquescence, the salt particles absorb water and dissolve slowly, which results in a solution with a salt concentration near the solubility limit. The positive control was set up to reduce the duration of osmotic stress that is caused by deliquescence by quickly adding 1 mL of anoxic water into the inner compartment.

### Methane measurements

The methane concentration in the headspace was determined with a gas chromatograph (Agilent Technologies, GC 6890), which was equipped with a Carbonplot capillary column (Ø 0.53 mm, 30 m length) and a flame ionization detector (FID). The injector and oven temperatures were 45 °C and the detector temperature was 250 °C. Helium was used a carrier gas. Calibration was performed with standards of the respective gases. Details of the methane analysis have been previously described^37^.

## Data Availability

All numerical data in this paper are provided in the figures and are also available in tabular form from the contact author upon request (schulze-makuch@tu-berlin.de).

## References

[1] Formisano V, Atreya S, Encrenaz T, Ignatiev N, Giuranna M (2004). Detection of methane in the atmosphere of Mars. Science.

[2] Webster CR (2018). Background levels of methane in Mars’ atmosphere show strong seasonal variations. Science.

[3] Krasnopolsky VA, Maillard JP, Owen TC (2004). Detection of methane in the martian atmosphere: evidence for life?. Icarus.

[4] Atreya SK, Mahaffy PR, Wong A-S (2007). Methane and related trace species on Mars: Origin, loss, implications for life, and habitability. Planet Space Sci.

[5] Möhlmann D (2005). Adsorption water-related potential chemical and biological processes in the upper Martian surface. Astrobiology.

[6] Möhlmann DT, Niemand M, Formisano V, Savijärvi H, Wolkenberg P (2009). Fog phenomena on Mars. Planet Space Sci.

[7] McEwen AS (2011). Seasonal flows on warm Martian slopes. Science.

[8] Dundas CM (2017). Granular flows at recurring slope lineae on Mars indicate a limited role for liquid water. Nat Geosci.

[9] Ojha Lujendra, Wilhelm Mary Beth, Murchie Scott L., McEwen Alfred S., Wray James J., Hanley Jennifer, Massé Marion, Chojnacki Matt (2015). Spectral evidence for hydrated salts in recurring slope lineae on Mars. Nature Geoscience.

[10] Bhardwaj A, Sam L, Martín-Torres FJ, Zorzano M-P, Fonseca RM (2017). Martian slope streaks as plausible indicators of transient water activity. Scientific reports.

[11] Osterloo MM (2008). Chloride-bearing materials in the southern highlands of Mars. Science.

[12] Clark BC, Kounaves SP (2016). Evidence for the distribution of perchlorates on Mars. International Journal of Astrobiology.

[13] Hecht M (2009). Detection of perchlorate and the soluble chemistry of martian soil at the Phoenix lander site. Science.

[14] Kounaves, S. *et al*. Wet Chemistry experiments on the 2007 Phoenix Mars Scout Lander mission: Data analysis and results. *Journal of Geophysical Research: Planets* **115** (2010).

[15] Rennó, N. O. *et al*. Possible physical and thermodynamical evidence for liquid water at the Phoenix landing site. *Journal of Geophysical Research: Planets* **114** (2009).

[16] Schulze-Makuch D (2018). Transitory microbial habitat in the hyperarid Atacama Desert. Proc Natl Acad Sci USA.

[17] Bajerski F, Wagner D (2013). Bacterial succession in Antarctic soils of two glacier forefields on Larsemann Hills, East Antarctica. FEMS microbiology ecology.

[18] Heinz J, Schirmack J, Airo A, Kounaves SP, Schulze-Makuch D (2018). Enhanced Microbial Survivability in Subzero Brines. Astrobiology.

[19] Heinz J (2019). Bacterial Growth in Chloride and Perchlorate Brines: Halotolerances and Salt Stress Responses of Planococcus halocryophilus. Astrobiology.

[20] Morozova D, Wagner D (2007). Stress response of methanogenic archaea from Siberian permafrost compared with methanogens from nonpermafrost habitats. FEMS Microbiol Ecol.

[21] Kral TA, Bekkum CR, McKay CP (2004). Growth of methanogens on a Mars soil simulant. Orig Life Evol Biosph.

[22] Schirmack J, Alawi M, Wagner D (2015). Influence of Martian regolith analogs on the activity and growth of methanogenic archaea, with special regard to long-term desiccation. Frontiers in microbiology.

[23] Serrano P, Alawi M, de Vera J-P, Wagner D (2019). Response of methanogenic archaea from Siberian permafrost and non-permafrost environments to simulated Mars-like desiccation and the presence of perchlorate. Astrobiology.

[24] Morozova D, Moeller R, Rettberg P, Wagner D (2015). Enhanced radiation resistance of Methanosarcina soligelidi SMA-21, a new methanogenic archaeon isolated from a Siberian permafrost-affected soil in direct comparison to Methanosarcina barkeri. Astrobiology.

[25] Sinha N, Kral TA (2013). Methanogen sensitivity to ultraviolet radiation: implications for life on Mars. Meteorit Planet Sci.

[26] Kral TA, Altheide ST (2013). Methanogen survival following exposure to desiccation, low pressure and martian regolith analogs. Planet Space Sci.

[27] Craig, P., Mickol, R., Archer, P. & Kral, T. Nontronite and montmorillonite as nutrient sources for life on Mars. 48th Lunar and Planetary Science Conference, March 24, 2017; The Woodlands, TX; USA, document # 20170002223 (2017).

[28] Wagner D, Schirmack J, Ganzert L, Morozova D, Mangelsdorf K (2013). Methanosarcina soligelidi sp. nov., a desiccation- and freeze-thaw-resistant methanogenic archaeon from a Siberian permafrost-affected soil. Int J Syst Evol Microbiol.

[29] Orosei R (2018). Radar evidence of subglacial liquid water on Mars. Science.

[30] Bryant MP, Boone DR (1987). Emended Description of Strain Mst (Dsm800t), the Type Strain of Methanosarcina-Barkeri. Int J Syst Bacteriol.

[31] Mah RA (1980). Isolation and characterization of *Methanococcus-mazei*. Curr Microbiol.

[32] Barker AH (1936). Studies upon the methane-producing bacteria. Archives of Microbiology.

[33] Schirmack J (2014). Methanobacterium movilense sp. nov., a hydrogenotrophic, secondary-alcohol-utilizing methanogen from the anoxic sediment of a subsurface lake. Int J Syst Evol Microbiol.

[34] Allen CC (1998). JSC Mars-1: a Martian soil simulant. Space.

[35] Morris RV, Golden DC, Bell JF, Lauer HV, Adams JB (1993). Pigmenting agents in Martian soils: inferences from spectral, Mossbauer, and magnetic properties of nanophase and other iron oxides in Hawaiian palagonitic soil PN-9. Geochim Cosmochim Acta.

[36] Hungate R (1969). Chapter IV a roll tube method for cultivation of strict anaerobes. Methods in Microbiology.

[37] Koch K, Knoblauch C, Wagner D (2009). Methanogenic community composition and anaerobic carbon turnover in submarine permafrost sediments of the Siberian Laptev Sea. Environmental Microbiology.

